# Stereotactic Body Radiotherapy (SBRT) in Very Limited-Stage Small Cell Lung Cancer (VLS-SCLC)

**DOI:** 10.3390/curroncol30010008

**Published:** 2022-12-22

**Authors:** Stéphanie L. Mercier, Sara M. Moore, Deborah Akurang, David Tiberi, Paul Wheatley-Price

**Affiliations:** 1 Department of Medicine, University of Ottawa, Ottawa, ON K1H 8L5, Canada; 2 Ottawa Hospital Research Institute, Ottawa, ON K1Y 4E9, Canada; 3 Department of Radiology, University of Ottawa, Ottawa, ON K1H 8L6, Canada

**Keywords:** SCLC, SBRT, SABR, limited stage

## Abstract

Small cell lung cancer (SCLC) is an aggressive neuroendocrine tumour with metastatic propensity. Stereotactic body radiation therapy (SBRT) is an emerging therapeutic option for SCLC, despite limited supporting evidence. By evaluating the use of SBRT in very limited stage (VLS) SCLC at our institution, we aimed to contribute to the existing knowledge in this area while establishing a basis for further research. We performed a retrospective review of all cases of VLS-SCLC treated with SBRT between 2013 and 2020. Baseline demographics, diagnostic, and treatment information were collected. The primary outcome was overall survival (OS). We identified 46 patients with pathologically confirmed VLS-SCLC; 25 were treated with SBRT, and the remainder received either surgery, conventional radiation therapy, chemotherapy, or palliative-intent therapy. After a median follow-up of 23.7 months, 44% of the patients had died; the median OS was of 24.4 months for the SBRT cohort and 67.0 months for the curative intent non-SBRT cohort. The difference in disease recurrence and survival between cohorts was underpowered and not statistically significant. Higher baseline ECOG and comorbidity was noted in the SBRT cohort.

## 1. Introduction

Lung cancer comprises a significant portion of cancer-related morbidity and mortality worldwide. In 2020, lung cancer was the most diagnosed cancer and the leading cause of cancer-related death in Canada [1]. Small cell lung cancer (SCLC), making up approximately 15% of diagnosed lung cancers [2], is an aggressive neuroendocrine tumour with high metastatic potential closely associated with tobacco exposure [3]. Given its rapid growth, SCLC generally responds well initially to treatment with either chemotherapy, radiotherapy or a combination, but typically has high recurrence rates [4]. While SCLC can be classified using the “Tumor, nodes and metastases” (TNM) staging system, it is more commonly classified as extensive stage or limited-stage using the Veterans Administration Lung Group (VALSG) 2-stage system [5]. Limited-stage (LS) disease, here defined as a tumour that can be encompassed by a single radical radiotherapy field, is “potentially curable”. Very limited-stage SCLC (VLS-SCLC) may be defined as T1-2, node negative disease. Approximately one third of SCLC cases are limited-stage at diagnosis. While developed to identify non-small cell lung cancer (NSCLC) at an early stage, the adoption of lung cancer screening with low-dose CT scan may result in more cases of SCLC diagnosed at limited-stage [6].

The gold standard curative-intent therapy for limited-stage SCLC is concurrent chemoradiation, often followed by prophylactic cranial irradiation (PCI), whereas surgery is only considered occasionally [4]. Despite limited data, stereotactic body radiation therapy (SBRT), also known as stereotactic ablative body radiotherapy (SABR), has been emerging as another treatment option in SCLC, recently making its way into treatment algorithms for early stage, node negative disease [7,8]. SBRT allows the delivery of focused high-intensity doses of radiation over fewer sessions than conventional radiotherapy, reducing complication rates and improving treatment adherence.

Our study aimed to evaluate the use of SBRT in very limited-stage SCLC at a single academic cancer centre.

## 2. Materials and Methods

We performed a retrospective chart review of all cases of very limited-stage small cell lung cancer (VLS-SCLC) treated at the Ottawa Hospital Cancer Centre between January 2013 and December 2020. The case list was defined through two methods: first, by identifying and filtering through all patients diagnosed with SCLC to identify those with very limited-stage disease; and second, by identifying all patients receiving SBRT to the lung and filtering for those with a diagnosis of SCLC. This was accomplished using two primary sources: data pulled from local contributions to the Canadian Small-Cell Lung Cancer Database (CASCADE) [9], and reports from the institution’s radiotherapy software. Further patient details and information were obtained through Epic, the institution’s electronic medical records system.

We included all patients aged 18 and older who were initially referred at time of diagnosis to a radiation oncologist for primary management of VLS-SCLC and who received any form of treatment to the primary tumour. Patients presenting locally advanced, distantly metastatic, or relapsed SCLC were excluded. 

Information was collected on baseline demographics (age, sex, smoking history and ECOG PS), staging investigations, tumour size, medical comorbidities, treatment information, and outcomes (disease relapse and vital status). A Charlson Comorbidity Index was calculated based on the medical comorbidities collected for each patient to further inform on baseline patient comorbid status. 

Two VLS-SCLC cohorts were created: those who were treated with SBRT, and those who received conventional therapies (conventional radiotherapy or surgery, with or without chemotherapy, and with or without PCI). We included patients who opted for palliative therapy or for no therapy in the second cohort to accurately represent the full population of VLS-SCLC. 

Details surrounding chemotherapy administration (including schedule, regimen, number of cycles administered, and reasons for discontinuation), use of PCI, and patient outcomes (including disease progression, relapse, and vital status at the end of the study period) were collected from medical oncology consult and progress notes.

Curative-intent therapy was defined as follows: (1) patients having received SBRT with or without chemotherapy; (2) a surgical procedure with curative intent; or (3) conventional radiotherapy greater than or equal to 40Gy with or without chemotherapy. 

Local relapse was defined as relapse within the original radiation field. Regional relapse was defined as any new regional disease in lymph nodes (N1-N3 per TNM staging guidelines). Distant relapse was defined as any evidence of metastatic disease (M1 per TNM staging guidelines). 

Statistical analysis was performed using the Statistical Package for the Social Sciences software version 23. The primary outcome was overall survival from the date of pathologic diagnosis of SCLC. Overall survival (OS) and disease recurrence comparisons were drawn between the SBRT group and non-SBRT patients who underwent curative-intent treatment only. OS was estimated using the Kaplan–Meier method. Patients were censored by the last date of known follow-up or investigation confirming that they were alive. Survival curves were compared using the log-rank test. Recurrence comparisons were performed using the Fine and Gray cumulative incidence function.

Secondary outcomes evaluated included a descriptive analysis of the cohorts, a comparison of treatment-related morbidity between our SBRT and non-SBRT cohorts undergoing curative-intent therapy, assessment of the number of patients whose cancer was diagnosed and treated according to the National Comprehensive Cancer Network (NCCN) guidelines, and assessment of local control rates (defined as a tumour being equal to or lesser than the size of the original tumour at the start of radiotherapy based on follow-up CT imaging). 

## 3. Results

### 3.1. Baseline Patient Characteristics

A total of 46 cases of very limited-stage SCLC were included in our study cohort. A total of 25 patients (54%) received SBRT (SBRT cohort) and 21 (46%) were either treated with surgery, conventional radiation therapy (RT), chemotherapy, a combination of the former, or palliatively (comparator cohort). The patients who received either palliative or no treatment (*n* = 3) were included for the purposes of enhancing cohort descriptions. The baseline characteristics of our cohort at the time of initial consultation with a radiation oncologist are represented in Table 1.

In our overall cohort, the median age at diagnosis was 73, 57% were women, and all patients were either former or current smokers. Eastern Cooperative Oncology Group performance status (ECOG PS) was 0–1 in 80%, and the median Charlson Comorbidity Index (CCI) was 6. 

Almost all patients underwent central nervous system (CNS) staging (45/46, 98%) with dedicated brain computed tomography (CT) or magnetic resonance imaging (MRI), and the majority had staging via PET scan (43/46, 94%). Only 11% of the total cohort (and none of the SBRT group) underwent invasive mediastinal staging. Pulmonary function tests were performed in 76% of patients. 

### 3.2. Treatment Details

Of the overall cohort, 94% underwent curative-intent therapy. The remaining 6% either underwent palliative chemoradiation (*n* = 1) or no treatment (*n* = 2). The primary curative-intent treatment details are outlined in Table 2.

All patients in the SBRT group (100%) had SBRT as their primary treatment modality. In the non-SBRT (comparator) group, 29% underwent surgical resection as their primary method of treatment. A further 52% underwent conventional chemoradiation therapy and 5% (*n* = 1) had radiation alone. A total of 10% opted for non-curative therapy; these are excluded from the following table.

The median time from diagnosis to treatment initiation (for patients receiving curative-intent therapy) was 31 days (range 0–166 days). Time until initiation of treatment was numerically shorter in the surgery cohort (median 10 days) versus the SBRT (median 29 days) and chemoradiation (median 39 days) cohorts.

Six patients underwent surgical resection as their primary treatment modality; half underwent lobectomy, and the other half underwent sub-lobar resection. All first had PET scans followed by fine needle aspirate biopsy of their lesions. Each biopsy was either suspicious for or diagnostic of cancer, though small cell lung cancer was only identified in three of the cases. The diagnosis of SCLC was discovered or confirmed through surgical pathology in the remaining three cases.

Curative-intent systemic therapy and PCI details are outlined in Table 3.

All patients were seen in consultation by a medical oncologist. Chemotherapy was administered to the majority (70%) of those in the curative-intent groups; the remainder did not receive chemotherapy due to factors including frailty, low functional status, medical comorbidities, and patient decision. The most common regimen was combination platinum-based (cisplatin or carboplatin) and etoposide therapy. A total of 56% of patients who received chemotherapy completed their course; toxicity (including poor tolerance due to side effects, end organ dysfunction, and febrile neutropenia) was the most common reason for early discontinuation. Irinotecan was used occasionally at a time of national etoposide shortage.

A total of 14 patients underwent PCI dosed at 25Gy over 10 fractions.

### 3.3. Survival

Patients were followed-up for a median of 23.7 months. At the time of analysis, 44% of patients had died. The median overall survival (OS) was of 67.0 months for the entire study cohort. The median OS was of 24.4 months in the SBRT cohort and of 67.0 months in the non-SBRT, curative-intent cohort. 

A sensitivity analysis was performed to examine the potential impact of delayed treatment on survival. Overall survival calculated from the date of treatment initiation showed similar results to the primary analysis, with median OS 65.9 months for non-SBRT, and 22.5 months for SBRT cohorts.

**Figure 1:**
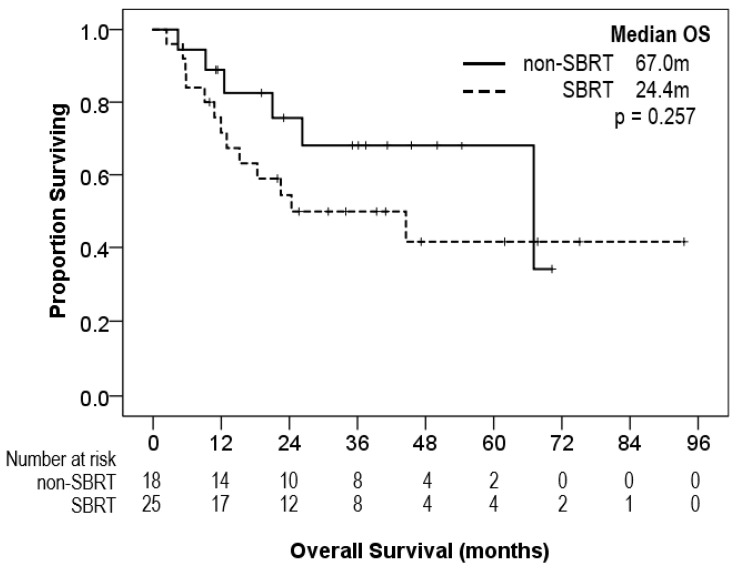
Overall survival (OS) comparing SBRT and curative-intent non-SBRT (surgery and conventional RT) cohorts.

**Figure 2:**
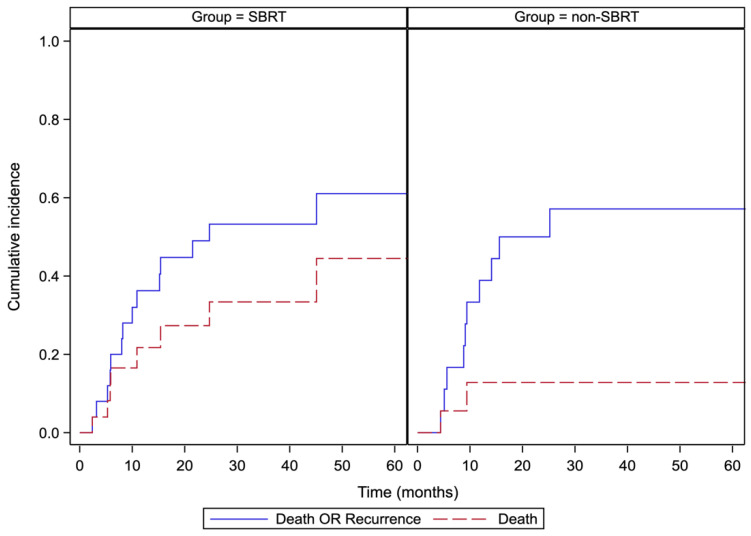
Cumulative incidence of death or recurrence versus death.

In the non-SBRT group, 44% experienced disease recurrence and 33% died from all causes. In contrast, only 24% of the SBRT group experienced disease recurrence; however, 52% were deceased from all causes. Figure 1 depicts survival curves. Figure 2 provides the incidence of disease recurrence or death in the cohorts receiving curative-intent therapy contrasted against death (*p* = 0.173).

## 4. Discussion

In this institutional review of the treatment of very-limited small cell lung cancer, we observed a variation in curative-intent treatment patterns. SBRT was used in over half of patients. Although the study was not powered to detect outcome differences between SBRT and non-SBRT cohorts, we observed numerically lower relapse rates among the SBRT cohort. However, more patients had died at endpoint for data collection in the SBRT cohort. The median overall survival of the non-SBRT group cohorts was numerically more than double that of the SBRT group. The divergence in survival between the two groups is primarily noted in the first 24 months of follow-up. Tumours were generally smaller in the SBRT and surgical groups compared to the CRT group.

Clinicians may have recommended the more easily delivered SBRT over conventional RT out of concern that their patients would not tolerate the latter well, suggesting that patient selection played a key role in our results. This is supported by a nominally higher Charlson Comorbidity Index (CCI) and a higher average ECOG score in the SBRT cohort relative to the CRT and surgery cohorts, which suggest that the SBRT group was more frail at baseline.

Due to differences in ECOG score, CCI, tumour size, and chemotherapy use between cohorts, we could not directly compare our treatment groups. Our results suggest that those in the non-SBRT group may have had more cancer-related mortality (possibly due to living longer overall and having fewer comorbidities), while those in the SBRT group may have died sooner due to factors related to a poorer baseline health status. We were unable to perform cancer-specific survival analyses due to incomplete cause of death data.

Per the 2022 NCCN guidelines, curative treatment options for very limited-stage (I-IIa) node-negative disease include: surgical resection with adjuvant systemic therapy; SBRT with adjuvant systemic therapy; or concurrent chemoradiation therapy (preferred to sequential radiation and systemic therapy) [8]. North American and European guidelines recommend chemotherapy in all curative-intent treatment regimens due to SCLC’s metastatic propensity [8,10]. In our overall cohort of 43 patients, only 31 received curative treatment as defined by the NCCN. The primary reasons cited for not receiving full curative therapy included patient decision, patient factors (including medical comorbidities and frailty), and the identification of metastatic disease partway into the initial treatment course. 

Eight of the twenty-five SBRT patients entirely forwent adjuvant chemotherapy, as did two of the six patients who underwent surgical resection. One patient originally destined for CRT completed her chemotherapy course but ultimately received lower-dose consolidative radiation therapy after restaging scans confirmed the presence of extensive stage disease. One further patient received conventional radiation therapy alone without chemotherapy. Overall, not receiving chemotherapy was by far the main reason the patients in our cohort did not receive the “full” curative treatment. Furthermore, among the patients who did initiate curative-intent chemotherapy, more than half did not complete their course due to toxicity or disease progression. This is not unexpected; in a real-world, comorbid population, it is common practice to give less chemotherapy than is recommended. The rate at which chemotherapy was given in our cohort was comparable to rates reported in other studies [11].

Sub-lobar, or wedge, resection is not a standard surgery for patients with SCLC. In our cohort, three patients had this procedure (of six total patients who had surgical resection as primary treatment). The reasons provided for selecting this procedure included preservation of functional lung tissue in the setting of marginal baseline pulmonary function and the peripheral location of a tumour lending itself well to a minimally invasive surgery.

SBRT has been considered a reasonable treatment option for limited-stage disease for many years now [12,13,14,15], despite a relative paucity of data (both retrospective and randomized) on the efficacy of SBRT in treating SCLC. At the time of writing, there exists only one published prospective phase II study demonstrating the efficacy of SBRT (in combination with platinum-based chemotherapy) at prolonging survival in patients with TNM stage 1, 2, and 3 SCLC [16]. Early retrospective studies and institutional reviews found favourable early progression free and overall survival using SBRT for SCLC [17,18,19,20]. These and other similar small clinical studies form the basis of the American Society for Therapeutic Radiology and Oncology (ASTRO) clinical guidelines. A recently published meta-analysis combining the results of 11 clinical studies and a total of 399 cases of T1-2N0M0 SCLC concluded that SBRT was effective for local control with minimal toxicity for inoperable early-stage node-negative disease [21]. The key findings in our study mirror those in the above studies: that SBRT is effective for local disease control with minimal toxicity. Conversely, a 2016 literature review noted that 3-year OS was superior in SBRT-treated patients compared to combined surgery and chemotherapy despite greater comorbidity in the former [22]; this survival effect was not reflected in our SBRT cohort (our comparison cohort, however, has very few surgical patients relative to CRT patients). Meanwhile, the limited existing data finding that SBRT may be used to treat early-stage operable NSCLC [23] have been extrapolated to early SCLC, but there is no published data directly comparing surgical resection to SBRT. Ultimately, due to its metastatic propensity, SCLC remains a systemic disease [24]; therefore, the precise method of local control may matter less than tolerance of systemic therapy. It has been well documented that chemotherapy improves outcomes in all patients with SCLC [25,26]; however, the rate of chemotherapy given has been consistently low in studies looking at SBRT for limited-stage SCLC [11], commonly due to tolerance concerns (as seen in the present study).

This study’s key limitations are secondary to its single-centre retrospective nature and small sample size. As this is an institutional review, we were limited to the number of patients who were treated for very limited-stage small cell lung cancer and therefore did not have statistical power to detect significant differences between treatment cohorts. An additional limitation is that some of the descriptive data collected relied on what information was reported in medical progress notes, which were variable in quantity and quality of content.

In the future, a larger multi-centre analysis could increase the sample size to a degree that correction or matching for differing baseline patient and disease characteristics would be feasible, thus enabling more robust comparisons of outcomes between SBRT and non-SBRT treated cohorts. Defining and refining patient selection criteria (such as comorbidity burden or tumour size and location) could provide the clinician with a clearer criterion, thereby facilitating patient selection for SBRT. Randomized controlled trials comparing SBRT to CRT and to surgical resection would help to better define the place of SBRT in the treatment algorithms for limited-stage SCLC. The method selected to achieve local control should be effective with minimal morbidity; future studies comparing these methods should focus on both points. Ongoing and future studies on immunotherapy may find this to be a more tolerable alternative to chemotherapy in the quest to achieve systemic control.

## 5. Conclusions

In conclusion, SBRT has made its way into official guidelines for the treatment of limited-stage non-small cell lung cancer, despite relatively scant data backing this practice. From our review of patients with very limited-stage small cell lung cancer between 2013 and 2020 at the Ottawa Hospital, SBRT has been used as frequently as conventional radiation therapy and surgery combined over this timeframe. We found a numerical difference in disease recurrence favouring patients treated with SBRT over non-SBRT (surgery or CRT). There was a numerical reduction in overall survival in the SBRT cohort, which may be related to greater medical frailty and non-cancer deaths in patients selected for SBRT. Our results suggest that higher baseline comorbidity and frailty may have biased clinicians towards selecting SBRT over other therapies. A larger study would be required to eliminate selection bias and power non-inferiority analyses comparing SBRT to other therapies.

## Figures and Tables

**Table 1:** Baseline characteristics of study population.

Characteristic		Overall; *n* = 46 (%)	SBRT; *n* = 25 (%)	Conventional RT; *n* = 12 (%)	Surgery; *n* = 6 (%)	Non-Curative Therapy; *n* = 3 (%)
Age	Median (range)	73 (55–89)	73 (55-89)	75.5 (62-84)	71.5 (63-89)	67 (66-76)
Sex	Male Female	20 (44%) 26 (56%)	11 (44%) 14 (56%)	5 (42%) 7 (58%)	3 (50%) 3 (50%)	1 (33%) 2 (67%)
Smoking history	Former Current	12 (26%) 34 (74%)	17 (68%) 8 (32%)	2 (17%) 10 (83%)	2 (33%) 4 (67%)	0 (0%) 3 (100%)
CCI	Median (range)	6 (3–12)	6 (3–12)	5.5 (4–11)	5 (5–6)	5 (4–6)
ECOG PS at diagnosis	0–1 ≥2	37 (80%) 9 (20%)	18 (72%) 7 (28%)	11 (92 %) 1 (8 %)	5 (83%) 1 (17%)	3 (100%) 0 (0%)
PET scan for staging	Yes No	43 (94%) 3 (6%)	23 (92%) 2 (8%)	12 (100%) 0 (0%)	6 (100%) 0 (0%)	2 (67%) 1 (33%)
CNS staging	MRI +/− CT CT None	34 (74%) 11 (24%) 1 (2%)	19 (76%) 6 (24%) 0 (0%)	8 (67%) 3 (25%) 1 (8%)	5 (83%) 1 (17%) 0 (0%)	2 (67%) 1 (33%) 0 (0%)
Invasive mediastinal staging	Mediastinoscopy EBUS None	3 (7%) 5 (11%) 38 (83%)	0 (0%) 0 (0%) 25 (100%)	0 (0%) 1 (8%) 11 (92%)	3 (50%) 2 (33%) 1 (17%)	0 (0%) 1 (33%) 2 (66%)
Primary tumor size	T1a <1 cm T1b 1–2 cm T1c 2–3 cm T2a 3–4 cm T2b 4–5 cm	3 (7%) 24 (52%) 9 (20%) 8 (17%) 2 (4%)	1 (4%) 16 (64%) 6 (24%) 2 (8%) 0 (0%)	0 (0%) 4 (33%) 3 (25%) 4 (33%) 1 (8%)	2 (33%) 3 (50%) 0 (0%) 1 (17%) 0 (0%)	0 (0%) 1 (33%) 0 (0%) 1 (33%) 1 (33%)

**Table 2:** Curative-intent treatment details.

(a) Conventional RT	
**RT Dose (Gy)/Fractions**	***n* = 12 (%)**
60/30	6 (50%)
60/20	2 (17%)
60/15	1 (8%)
others	3 (25%)
(b) SBRT	
**RT Dose/Fractions**	***n* = 25 (%)**
60/8	4 (16%)
60/5	11 (44%)
54/3	8 (32%)
50/5	1 (4%)
48/4	1 (4%)
**RT Schedule**	
Daily	8 (32%)
Every 3 days	17 (68%)
(c) Surgery	
**Surgery Type**	***n* = 6 (%)**
Lobectomy	3 (50%)
Sub-lobar resection	3 (50%)

**Table 3:** Systemic therapy and PCI.

Treatment Details		SBRT; *n* = 25 (%)	Conventional RT; *n* = 12 (%)	Surgery; *n* = 6 (%)
Systemic therapy use	Yes	17 (68%)	11 (92%)	4 (67%)
	No	8 (32%)	1 (8%)	2 (33%)
Systemic therapy schedule	Sequential (neoadjuvant)	5 (29%)	1 (9%)	1 (25%)
	Concurrent	1 (6%)	10 (91%)	0
	Sequential (adjuvant)	11 (65%)	0	3 (75%)
Chemotherapy regimen	Cis * + etop **	9 (53%)	9 (82%)	2 (50%)
	Carbo *** + etop	4 (24%)	2 (18%)	0
	Carbo + irinotecan	1 (6%)	0	1 (25%)
	CAV ****	2 (12%)	0	1 (25%)
	Etoposide	1 (6%)	0	0
Chemotherapy: # of cycles administered	1	5 (29%)	0	1 (25%)
	2	2 (12%)	0	0
	3	1 (6%)	2 (18%)	1 (25%)
	4	6 (25%)	3 (27%)	2 (50%)
	≥5	3 (18%)	5 (45%)	0
	Unknown	0	1 (9%)	0
Reason for chemotherapy discontinuation	Course complete	8 (32%)	8 (73%)	2 (50%)
	Progression	0	0	0
	Toxicity	7 (28%)	2 (18%)	2 (50%)
	Death	1 (4%)	0	0
	Other	1 (4%)	1 (9%)	0
PCI	Yes	6 (24%)	6 (50%)	2 (33%)
	No	19 (76%)	6 (50%)	4 (67%)

* Cisplatin. ** Etoposide. *** Carboplatin. **** Cyclophosphamide, doxorubicin and vincristine.

## Data Availability

The data presented in this study are available on request from the corresponding author. The data are not publicly available due to privacy restrictions.
